# Synthesis and Properties of α-Mangostin and Vadimezan Conjugates with Glucoheptoamidated and Biotinylated 3rd Generation Poly(amidoamine) Dendrimer, and Conjugation Effect on Their Anticancer and Anti-Nematode Activities

**DOI:** 10.3390/pharmaceutics14030606

**Published:** 2022-03-10

**Authors:** Joanna Markowicz, Stanisław Wołowiec, Wojciech Rode, Łukasz Uram

**Affiliations:** 1 Faculty of Chemistry, Rzeszów University of Technology, 6 Powstancow Warszawy Ave, 35-959 Rzeszów, Poland; luram@prz.edu.pl; 2 Medical College, Rzeszów University, 1a Warzywna Street, 35-310 Rzeszów, Poland; swolowiec@ur.edu.pl; 3 Nencki Institute of Experimental Biology, 3 Pasteur Street, 02-093 Warsaw, Poland; w.rode@nencki.edu.pl

**Keywords:** α-mangostin conjugate, vadimezan conjugate, PAMAM G3 dendrimer, cytotoxicity, proliferation, glioblastoma, squamous cell carcinoma, human fibroblasts, *Caenorhabditis elegans*

## Abstract

α-Mangostin and vadimezan are widely studied potential anticancer agents. Their biological activities may be improved by covalent bonding by amide or ester bonds with the third generation poly(amidoamine) (PAMAM) dendrimer, substituted with α-D-glucoheptono-1,4-lactone and biotin. Thus, conjugates of either ester- (**G3^gh4B5V^**) or amide-linked (**G3^2B12gh5V^**) vadimezan, and equivalents of α-mangostin (**G3^gh2B5M^** and **G3^2B12gh5M^**, respectively), were synthesized, characterized and tested in vitro against cancer cells: U-118 MG glioma, SCC-15 squamous carcinoma, and BJ normal human fibroblasts growth, as well as against *C. elegans* development. α-Mangostin cytotoxicity, stronger than that of Vadimezan, was increased (by 2.5–9-fold) by conjugation with the PAMAM dendrimer (with the amide-linking being slightly more effective), and the strongest effect was observed with SCC-15 cells. Similar enhancement of toxicity resulting from the drug conjugation was observed with *C. elegans*. Vadimezan (up to 200 µM), as well as both its dendrimer conjugates, was not toxic against both the studied cells and nematodes. It showed an antiproliferative effect against cancer cells at concentrations ≥100 µM. This effect was significantly enhanced after conjugation of the drug with the dendrimer via the amide, but not the ester bond, with **G3^2B12gh5V^** inhibiting the proliferation of SCC-15 and U-118 MG cells at concentrations ≥4 and ≥12 μM, respectively, without a visible effect in normal BJ cells. Thus, the drug delivery system based on the PAMAM G3 dendrimer containing amide bonds, partially-blocked amino groups on the surface, larger particle diameter and higher zeta potential can be a useful tool to improve the biological properties of transported drug molecules.

## 1. Introduction

Many natural and synthetic compounds with anticancer potential are not active enough in terms of bioavailability and full exploitation of their biological properties. Xanthones, presenting examples of such compounds, are polyphenolic heterocyclic compounds that can be isolated from mangosteen rind or semi/fully synthesized [1]. α-Mangostin (αM), the main representative of the xanthones family, exhibits anticancer activity through various molecular mechanisms, the most important of which are the induction of mitochondria-mediated apoptosis and the inhibition of proliferation, migration, invasion, and angiogenesis [2,3,4,5,6]. These activities were confirmed with in vitro and in vivo models of brain [7], pancreatic [8], liver [9], cervical [10], prostate [11] and colorectal [12] cancers. Recently, αM co-administered with doxorubicin was reported to be effective in reducing cell stemness in luminal breast cancer [13].

Another xanthone, vadimezan (5,6-dimethylxanthenone-4-acetic acid, DMXAA, ASA404), is an analogue of flavone acetic acid, considered one of the most promising antivascular agents. Direct and indirect mechanisms of action have been proposed for Vadimezan (V). A direct mechanism of V action includes the induction of apoptosis in endothelial cells, which causes hemorrhagic necrosis and ischemia in tumor tissues and, consequently, a specific and irreversible destruction of established tumor vasculatures, resulting in the complete blockade of tumor blood flow. Indirect mechanisms of V action include activation of the innate immune system, which stimulates production of inflammatory cytokines (such as TNF and IL-6), activation of NFκB and p38 (MAPK), production of nitric oxide, and reduction of tumor energetics and membrane turnover [14,15]. Later, the molecular target of V was discovered—the stimulator-of-interferon-genes (STING) protein that plays a central role in the innate immune system response. Although due to the satisfying results of in vivo studies, V has undergone several clinical trials as a single agent or in combination with taxanes, as well as in the context of carboplatin-based chemotherapy, the results of phase III clinical trials were disappointing [16]. One of the reasons for the lack of efficacy is the specificity of V to stimulate only murine STING protein but not the human STING [17]. Despite the failure of V to act as a STING agonist, attempts are made to re-develop V alone or in combination with properly targeted delivery systems. Liu et al. [18] introduced the hSTING mutant, STING^S162A/G230I/Q266I^, to reignite STING activity in Merkel cell carcinoma (MCC), where it is completely silenced. Since this hSTING mutant is highly sensitive to V, the delivery of STING^S162A/G230I/Q266I^ to MCC cells and treatment with V stimulated downstream antitumor cytokine production, T cell migration, and T cell activation in vitro. Boron difluoride dipyrromethene (BODIPY)-vadimezan conjugate was synthesized and enwrapped in mPEG-PPDA polymer brushes to obtain a highly-efficient type I photosensitizer for photodynamic therapy of hypoxic-and-metastatic tumors [19].

The employment of drug nanocarriers can improve water solubility and biodistribution of a drug transported in an organism. An ideal nanocarrier should present certain features, among which the most important are hydrophilicity, biocompatibility, and non-immunogenicity. Furthermore, it should be easy to conjugate or encapsulate the transported drug, as well as a proper ligand or targeting moiety, which can direct the drug to selected tissues and cells compartments [20]. Poly(amidoamine) (PAMAM) dendrimers meet the above-mentioned requirements. They are three-dimensional and hyperbranched polymers, attractive for drug delivery purposes [21,22]. Due to their spherical shape and easy-to-modify surface amine groups, they allow for the sustained release of the drug. This effect can be achieved by selecting an appropriate linkage between drug and nanocarrier. The use of different types of drug linkages can result in different drug release sites and rates. The mechanism of drug release may involve enzymatic or pH-dependent hydrolysis. Amide and ester bonds are most commonly used for the conjugation of small drugs to polymers, whereas other linkages, for example, disulfide bonds, have also been demonstrated. Amide bonds are generally stable towards various reaction conditions (acidic and basic), high temperature, and the presence of other chemicals. Additionally, they are the least reactive and their degradation is very slow as the amide bond is stabilized by the partial double bond [23]. However, Luo et al. [24] proved that in a multifunctional enveloped nano-carrier with PEG-PLL(DMA) (poly(ethylene glycol)-blocked-2,3-dimethylmaleic anhydride-modified poly(L-lysine) on its surface, the hydrolysis of amide linkages between amines and DMA occurred at acidic pH, while at physiological pH, amide bonds remained stable. The main enzymes responsible for amide hydrolysis are serine and cysteine hydrolases [25]. Ester linkages are generally easy to hydrolyze and the esterases play a major role in their enzymatic degradation. Furthermore, esterase hydrolysis represents an interesting potential strategy for targeted therapy of cancers that overexpress esterases, or which express esterases with different specificities [26,27]. Kurtoglu et al. compared ibuprofen release profiles from ester- and amide-bonded conjugates of the PAMAM G4 dendrimer and found that while the hydrolysis of ester bonds was pH-dependent, the amide conjugates were very stable at all pH buffers, suggesting the drug release to be caused by enzymatic cleavage [28].

We have previously used biotinylated and glucoheptoamidated 3rd generation poly(amidoamine) dendrimer (PAMAM G3) to covalently attach αM by amide bond (**G3^12gh2B5M^**) [29] and demonstrated that drug coupling with the non-toxic vehicle significantly increased its cytotoxicity and improved other biological activities, such as pro-apoptotic and anti-proliferative in vitro, as well as nematocidal in vivo. The goal of this paper was the design, synthesis and characterization of three biotinylated and glucoheptoamidated PAMAM G3 dendrimer conjugates: (i) with ester-linked αM (**G3^gh2B5M^**), (ii) with ester-linked V (**G3^gh4B5V^**) and (iii) with amide-linked V (**G3^12gh2B5V^**) and investigation of the influence of their size, zeta potential, and the type of chemical bond between drugs and carrier on their biological activity in vivo and in vitro. Biotin was used as a targeting agent, in view of certain cancer cells having been demonstrated to overexpress biotin receptors and transporters [30]. Moreover, biotinylation of PAMAM dendrimers significantly increases cellular uptake and accumulation of these nanoparticles compared to non-biotinylated ones [31,32,33,34]. Biotinylation allows also PAMAM dendrimers to cross the blood-brain barrier more effectively, which is essential in the treatment of central nervous system tumors, for example, gliomas [35]. Glucoheptoamidation was performed to increase the conjugate solubility and block the amine groups present on the dendrimer surface that are responsible for the cytotoxicity of the native PAMAM G3 dendrimers [36]. The NMR and DLS analyses of the obtained conjugates were performed. The anti-cancer activity of conjugates was studied on human grade IV glioma cells (U-118 MG), human squamous carcinoma cells (SCC-15), compared to normal human fibroblasts (BJ). *Caenorhabditis elegans* nematode, widely used in nano-sciences to study genotoxicity, neurotoxicity and impact on reproduction [37], was used to study nematocidal activity in vivo.

## 2. Materials and Methods

### 2.1. Reagents

α-Mangostin (αM, purity ≥ 98% (HPLC)) was purchased from Aktin Chemicals, Inc. (Chengdu, China) and vadimezan (V, DMXAA, purity > 98%) was obtained from DC Chemicals (Shanghai, China). Ethylenediamine, methyl acrylate, D-glucoheptono-1,4-lactone (GHL), succinic anhydride (SucAnh), 2-chloro-1-methylpyridinium iodide (Mukaiyama reagent), 4-dimethylaminopyridine (DMAP), biotin, dimethyl sulfoxide (DMSO) and other reagents used in chemical syntheses were provided by Merck KGaA (Darmstadt, Germany). Spectra/Por^®^ 3 RC dialysis membrane (cellulose, MW_cutoff_ = 3.5 kDa) was purchased in Carl Roth GmbH & Co. KG (Karlsruhe, Germany).

### 2.2. Biochemical Reagents, Cell Lines and Materials

Human cancer cell lines: glioblastoma (U-118 MG) and squamous cell carcinoma (SCC-15), human normal fibroblast cell line (BJ), Eagle’s Minimum Essential Medium (EMEM) and fetal bovine serum (FBS) used for supplementation of this medium were obtained from the American Type Culture Collection (ATCC, Manassas, VA, USA). Dulbecco’s Modified Eagle’s Media (DMEM and DMEM/F-12) and fetal bovine serum (FBS) were purchased from Corning Inc. (New York, NY, USA). Penicillin and streptomycin solution, phosphate-buffered saline (PBS) with and without magnesium and calcium ions, and Hoechst 33,342 were provided by Thermo Fisher Scientific Inc. (Waltham, MA, USA). Trypsin-EDTA solution, hydrocortisone, 0.33% neutral red solution, XTT sodium salt, phenazinemethosulfate (PMS), 0.4% trypan blue solution, dimethylsulfoxide (DMSO) for molecular biology, 5-Fluoro-2’-deoxy-uridine (FUdR), and other chemicals and buffers were purchased from Merck KGaA (Darmstadt, Germany). Cell culture dishes and materials were from Corning Inc. (New York, NY, USA), Greiner (Kremsmünster, Austria), or Nunc (Roskilde, Denmark). All reagents used to culture and synchronize *C. elegans* nematodes were supplied by Sigma-Aldrich (Saint Louis, MO, USA) or Carl Roth GmbH & Co. KG (Karlsruhe, Germany).

### 2.3. Syntheses of Dendrimer Conjugates with Ester-Bonded Biotin and α-Mangostin or Vadimezan

#### 2.3.1. Synthesis of Fully Glucoheptoamidated PAMAM G3

**Scheme 1:**
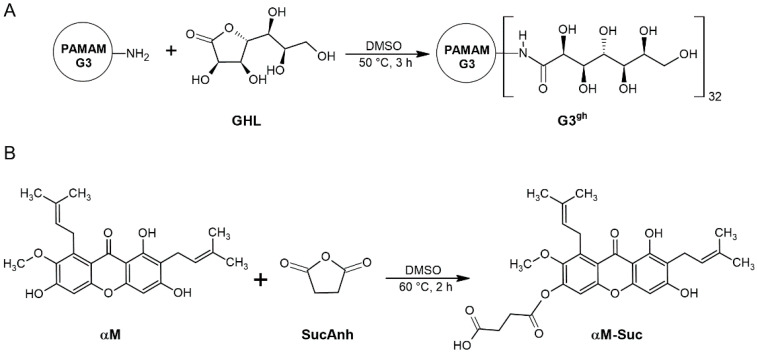
The conversion of (**A**) PAMAM G3 dendrimer into fully glucoheptoamidated derivative **G3^gh^** and (**B**) α-mangostin into carboxy-terminated derivative αM-Suc using succinic anhydride.

PAMAM G3 dendrimer was obtained according to Tomalia’s protocol [38] and was converted into fully glucoheptoamidated derivative G3^32gh^ by reaction with an excess of α-D-glucoheptono-1,4-lactone (GHL) in DMSO (Scheme 1A) as described earlier [39] on a scale of 95 μmoles. Thus, the PAMAM G3 (661 mg, 95.6 μmoles) was dissolved in 5 mL of DMSO. To this solution a two-fold excess of GHL (1273 mg, 6118 μmoles) was added in portions with vigorous stirring. The mixture was heated at 50 °C for 12 h, transferred to a dialytic bag and dialyzed for 3 days against water. Water was removed by vacuum rotary evaporation and a solid residue was dried overnight under high vacuum (2 mbars). The final product (**G3^gh^**) was obtained in 35.4% yield (459 mg, 33.83 μmoles, MW = 13,568 g mol^−1^). This compound was then used as a core dendrimer to attach covalently biotin and α-mangostin or vadimezan via ester bonds. Five µmoles of **G3^gh^** were used as stock solution (12.7 mM in water) for biological studies to evaluate the cytotoxicity of the dendrimeric nanocarrier.

#### 2.3.2. Synthesis of Biotinylated G3^gh^—α-Mangostin Conjugate

The α-mangostin (αM) was converted into a 6-succinate derivative with succinic anhydride (Scheme 1B) as it was performed previously [39].

46.3 mg (112 µmoles) of αM was dissolved in 2 mL of DMSO and then succinic anhydride (17 mg, 168 µmoles) was added. The solution was heated at 60 °C for 2 h and used later to conjugate αM to dendrimer as follows: to the G3^gh^ dendrimer (300 mg, 22 µmoles) solution in 2 mL of DMSO, 11 mg of biotin (44 µmoles) was added followed by dropwise addition of αM-Suc in DMSO. Then, DMAP (57.2 mg, 468 µmoles) and Mukaiyama reagent (59.8 mg, 234 µmoles) were added and the mixture was heated at 50 °C for 18 h. Afterwards, the solution was transferred into a dialytic tube (cellulose, MW_cutoff_ = 3.5 kDa) and dialyzed for 3 days against water (six times 3 L). Water was removed by rotary evaporation and remained solid was dried under high vacuum for 12 h. 201 mg of final product was obtained and dissolved in 2.5 mL of DMSO-d_6_. The product was identified by ^1^H-NMR spectroscopy as **G3^gh2B5M^** and stored as a stock solution of 4.9 mM concentration. The isolated yield of the product was 55.5% (12.2 µmoles, MW_calc_ = 16,451 g mol^−1^).

**Figure 1:**
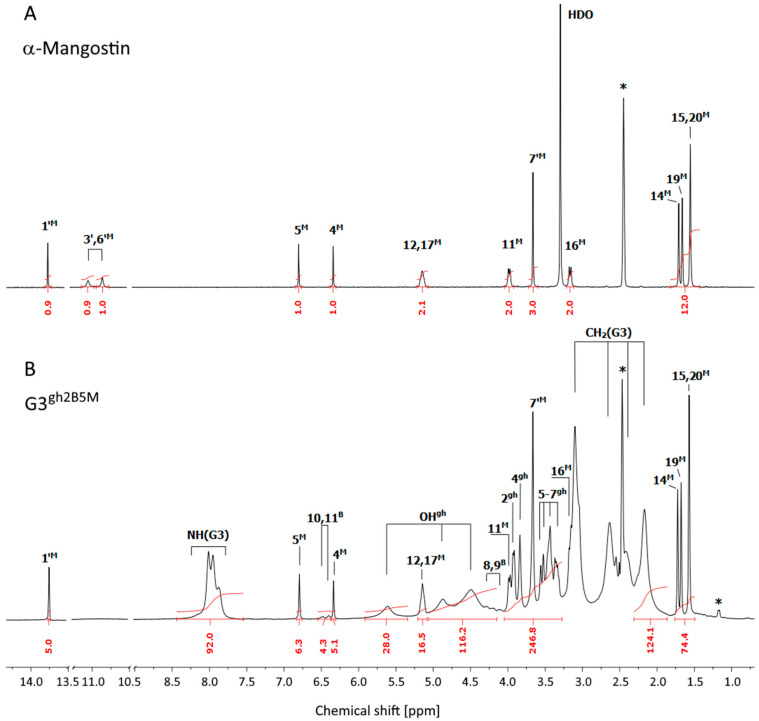
The ^1^H NMR spectra of α-mangostin (**A**) and **G3^gh2B5M^** conjugate (**B**) in DMSO-d_6_. The residual solvent peak at 2.5 ppm and impurity resonances are marked with asterisks *. The PAMAM G3 core dendrimer resonances are labeled as CH_2_(G3) and NH(G3) in spectrum B. The resonances of α-mangostin, biotin, and glucoheptoamide are labeled by locants with ^M^, ^B^, and ^gh^ upper indexes, respectively, according to the atom numbering in Scheme 2. The 1A spectrum was taken from [29].

**Scheme 2:**
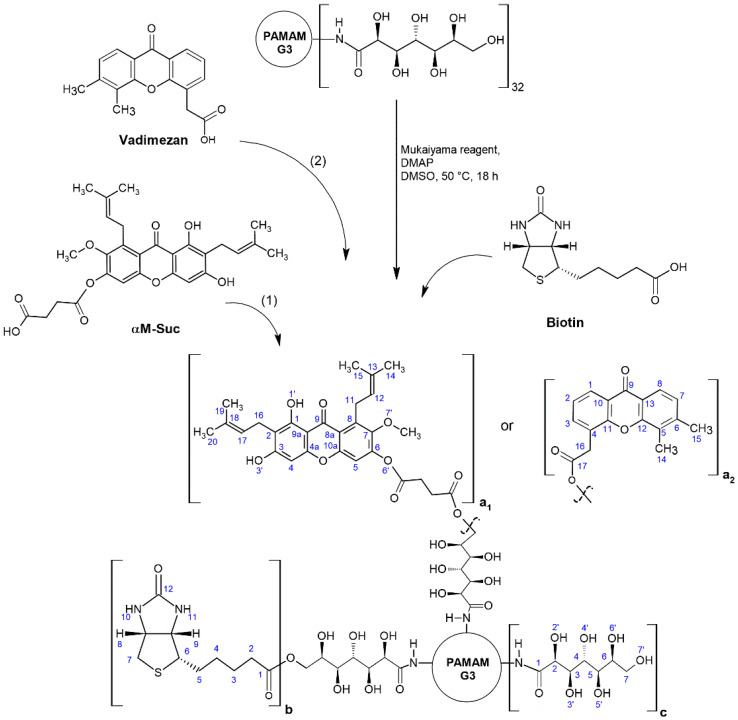
Synthesis of PAMAM G3^gh^ dendrimer conjugates with α-mangostin (1) or vadimezan (2), and biotin attached via ester bond: (1) **G3^gh2B5M^** (a_1_ = 5, a_2_ = 0, b = 2, c = 25), (2) **G3^gh4B5V^** (a_1_ = 0, a_2_ = 5, b = 4, c = 23).

Analytical data:

^1^H NMR (DMSO-d_6_): chemical shift [ppm] (intensity, multiplicity, assignment): **G3^gh2B5M^** (for atom numbering see Scheme 2, for spectrum see Figure 1B): 13.73 ([5H], s, 1′^M^); 6.79 ([5H], s, 5^M^); 6.34 ([5H], s, 4^M^); 5.15 ([10H], overlapped t, 12^M^, 17^M^); 3.99 ([10H], m, 11^M^); 3.69 ([15H], s, 7′^M^); 3.19 ([10H], 16^M^); 1.69 ([60H], overlapped s, 14^M^, 15^M^, 19^M^, 20^M^); PAMAM G3 resonances: 7.93 ([60+32H], m, NH(G3)); 3.13-2.20 ([484H], CH_2_(G3)); biotin resonances: 6.48 and 6.40 ([4H], s and s, 10^B^ and 11^B^); 4.29 and 4.12 ([4H], s and s, 8^B^ and 9^B^); glucoheptoamide OH resonances: 5.62, 5.07-4.16 (overlapped s, OH^gh^); CH_2_ resonances: 3.94 ([32H], bm, 2^gh^); 3.86 ([32H], bm, 4^gh^); 3. 69 (m, 3^gh^ overlapped with 7′^M^); 3.60-3.30 ([128H], overlapped s and m, 5-7^gh^). For detailed peak assignments see COSY spectrum (Figure S1, Supplementary materials), HSQC/HMBC combined spectra (Figure S2), and Table S1.

#### 2.3.3. Synthesis of Biotinylated G3^gh^—Vadimezan Conjugate

The **G3^gh^** dendrimer (172 mg, 12.7 µmoles) was dissolved in 3 mL of DMSO. In this solution, vadimezan (20.0 mg, 76 µmoles) and biotin (18.6 mg, 76 µmoles) were dissolved. Then DMAP (74.4 mg, 608 µmoles) was added followed by stepwise addition of Mukaiyama reagent (77.8 mg, 304 μmoles) into solution and heated at 50 °C for 18 h. The mixture was placed in a dialytic bag (cellulose, MW_cutoff_ = 3.5 kDa) and dialyzed against water for 4 days (8 times 3L). Water was evaporated at 50 °C and remaining solid (71 mg) was dissolved in 650 µL DMSO-d_6_. The ^1^H NMR spectrum was taken at room temperature and the average stoichiometry of conjugate was determined based on integral integration of biotin (B) and vadimezan (V) resonances versus G3^gh^ reference resonances (vide infra), as **G3^gh4B5V^**. The concentration of **G3^gh4B5V^** in DMSO-d_6_ was 6.9 mM. The isolated yield of the product was 35% (71 mg, 4.5 μmoles, MW_calc_ = 15,795 g mol^−1^).

**Figure 2:**
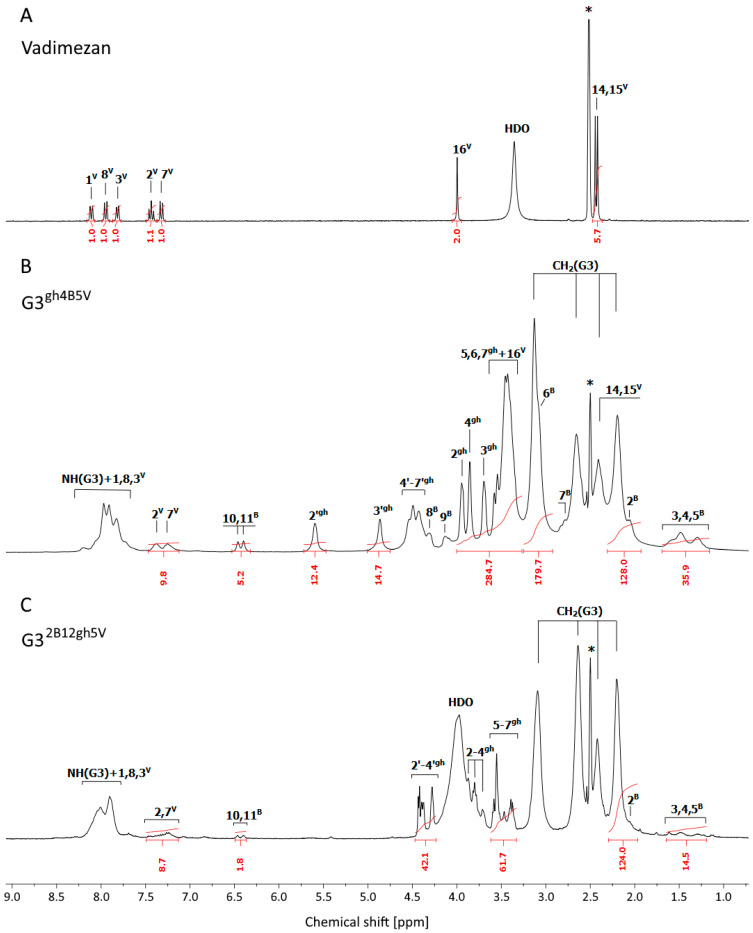
The ^1^H NMR spectra of vadimezan (**A**), **G3^gh4B5V^** (**B**) and **G3^2B12gh5V^** (**C**) conjugates in DMSO-d_6_. The residual solvent peak at 2.5 ppm is marked with asterisks *. The PAMAM G3 core dendrimer resonances are labeled as CH_2_(G3) and NH(G3) in spectrum B and C. The resonances of vadimezan, biotin, and glucoheptoamide are labeled by locants with ^V^, ^B^, and ^gh^ upper indexes, respectively, according to the atom numbering in Scheme 2 and Scheme 3.

Analytical data:

^1^H NMR (DMSO-d_6_): chemical shift [ppm] (intensity, multiplicity, assignment): **G3^gh4B5V^** (for atom numbering see Scheme 2, for spectrum see Figure 2B): 8.20-7.72 ([75H], m, NH(G3)+1,8,3^V^); 7.38 and 7.26 ([10H], overlapped t and d, 2^V^ and 7^V^); 2.37 ([30H], overlapped, 14^V^,15^V^); biotin resonances: 6.46 and 6.40 ([4H] and [4H], overlapped s, 10^B^ and 11^B^); 4.31 and 4.13 ([4H] and [4H], 8^B^ and 9^B^); 2.82 and 2.78 ([4H] and [4H], 6^B^ and 7^B^); 2.05 ([8H], 2^B^); 1.56-1.29 ([24H], overlapped m, 3^B^, 4^B^, 5^B^); gh OH resonances: 5.60 ([32H], s, 2′^gh^); 4.86 ([32H], s, 3′^gh^); 4.54-4.43 ([128H], m, 4′-7′^gh^); gh CH_2_ resonances: 3.94 ([32H], bs, 2^gh^); 3.85 ([32H], bs, 4^gh^); 3.69 ([32H], bs, 3^gh^); 3.58-3.43 ([128H], m, 5^gh^, 6^gh^, 7^gh^); PAMAM G3 CH_2_ broad resonances: 3.13-2.19 ([484H], CH_2_(G3)).

For detailed peak assignments see COSY spectrum (Figure S3), HSQC/HMBC combined spectra (Figure S4), and Table S1. For comparison the vadimezan spectrum (Figure 2A) was recorded and the resonances were assigned based on 2-D COSY and HSQC/HMBC spectra: 8.09 ([1H], d, 1^V^); 7.92 ([1H], d, 8^V^); 7.79 ([1H], d, 3^V^); 7.41 ([1H], t, 2^V^); 7.30 ([1H], d, 7^V^); 3.98 ([2H], s, 16^V^); 2.41 ([6H], overlapped s, 14,15^V^).

### 2.4. Syntheses of Dendrimer Conjugates with Amide-Bonded Biotin, GHL and α-Mangostin or Vadimezan

The detailed synthetic pathway of biotinylated and half-glucoheptoamidated dendrimer PAMAM G3 with α-mangostin (**G3^2B12gh5M^**) and its chemical characterization were described previously [29].

#### Synthesis of Biotinylated and Half-Glucoheptoamidated G3 with Vadimezan

**Scheme 3:**
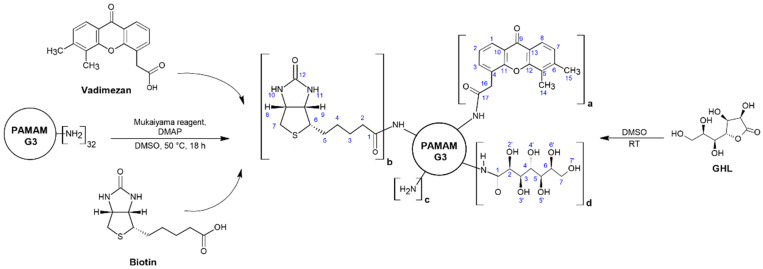
Synthesis pathway of PAMAM G3 dendrimer substituted with vadimezan, biotin and α-D-glucoheptono-1,4-lactone attached via amide bond: **G3^2B12gh5V^** (a = 5, b = 2, c = 13, d = 12).

Vadimezan (53 mg, 188 μmoles) and biotin (23 mg, 94 μmoles) were dissolved in 4 mL of DMSO. To this solution 51.7 mg (423 μmoles) of DMAP and 72 mg (282 μmoles) of Mukaiyama reagent were added, stirred until completely dissolved, and left at room temperature for 1 h. This mixture was added dropwise to a PAMAM G3 dendrimer (216 mg, 31.3 μmoles) solution in 2 mL of DMSO, followed by heating at 50 °C for 18 h. The reaction mixture was worked-up by dialysis, evaporation of solvent, and drying under high vacuum as before. The obtained solid was slightly soluble in DMSO. The ^1^H-NMR analysis allowed us to identify the product as G3^2B5V^ and the isolated yield was 35.5% (87 mg, 11.1 μmoles, MW_calc_ = 7810 g mol^−1^). Finally, glucoheptoamidation of the remaining amine groups was performed by reaction of G3^2B5V^ with GHL (Scheme 3). Thus, to G3^2B5V^ solution (87 mg, 11.1 μmoles) in 2.25 mL of DMSO-d_6_, GHL (28 mg, 133.6 μmoles) was added stepwise with vigorous stirring at RT. The product became soluble in DMSO. The ^1^H-NMR measurement was performed and allowed the determination of average stoichiometry of the conjugate as **G3^2B12gh5V^**. Based on a calculated average molecular weight (MW_calc_ = 11,306 g mol^−1^) the stock solution of this conjugate was estimated as 3.3 mM.

Analytical data:

^1^H NMR (DMSO-d_6_): chemical shift [ppm] (intensity, multiplicity, assignment): **G3^2B12gh5V^** (for atom numbering see Scheme 3, for spectrum see Figure 2C): 8.20-7.69 ([73H], m, NH(G3)+1,8,3^V^); 7.33 ([10H], overlapped t and d, 2^V^ and 7^V^); biotin resonances: 6.46 and 6.40 ([2H] and [2H], overlapped s, 10^B^ and 11^B^); 2.05 ([4H], 2^B^); 1.61-1.29 ([12H], 3^B^, 4^B^, 5^B^); gh OH resonances: 4.43-4.28 ([36H], m, 2′-4′^gh^); gh CH_2_ resonances: 3.87-3.71 ([36H], m, 2-4^gh^); 3.58-3.37 ([48H], m, 5-7^gh^); PAMAM G3 CH_2_ broad resonances: 3.09-2.20 ([484H], CH_2_(G3)).

### 2.5. NMR Spectroscopy

The 1-D ^1^H and ^13^C NMR spectra and 2-D ^1^H-^1^H correlations spectroscopy (COSY), ^1^H-^13^C heteronuclear single quantum correlation (HSQC), and heteronuclear multiple bond correlation (HMBC) spectra were obtained with Bruker 300 MHz instrument (Rheinstetten, Germany) at the University of Rzeszow (College of Natural Sciences).

### 2.6. Conjugates Size and ζ Potential Measurements

ζ potential and size of **G3^gh2B5M^**, **G3^12gh2B5M^**, **G3^gh4B5V^** and **G3^12gh2B5V^** were estimated with the dynamic light scattering technique at pH 5 (0.05 M acetate buffer) and pH 7 (water) using the Zetasizer Nano instrument (Malvern, UK). Measurements were performed for 1 mg/mL samples (0.06–0.1 mM solutions).

### 2.7. Biological Studies

#### 2.7.1. Cell Cultures

Human cancer cell lines: U-118 MG (glioblastoma multiforme, grade IV) and SCC-15 (squamous cell carcinoma) were grown in DMEM and DMEM/F-12 with hydrocortisone (400 ng/mL), respectively. Normal human skin fibroblasts (BJ) were cultured in EMEM. Growth media were supplemented with heat-inactivated 10% FBS and 100 U/mL penicillin and 1% streptomycin solution. Cells were cultured at 37 °C in a humidified 95% air/5% CO_2_ with growth media changed every 2–3 days. Cells were passaged at 75%–85% confluence using 0.25% trypsin–0.03% EDTA in PBS (calcium and magnesium ions free). The morphology of cells was observed under a Nikon TE2000S Inverted Microscope with phase contrast (Tokyo, Japan). The number and viability of cells were estimated by a trypan blue exclusion test using an Automatic Cell Counter TC20 (BioRad Laboratories, Hercules, CA, USA) or a Neubauer chamber. All assays were performed in triplicates in three independent experiments. The working solutions of the synthesized dendrimer conjugates and the drugs alone were prepared from stock solutions in cell culture media with an adjusted concentration of DMSO. Control samples with non-treated cells in a complete culture medium with adjusted DMSO concentration were included in all biological tests.

#### 2.7.2. Cytotoxicity

The cytotoxicity of αM, V, and their conjugates with PAMAM G3 modified dendrimer (**G3^gh2B5M^**, **G3^2B12gh5M^**, **G3^gh4B5V^** and **G3^2B12gh5V^**) was examined with neutral red (NR) uptake assay or XTT reduction assay. BJ, U-118 MG and SCC-15 cells were seeded in flat, clear bottom 96-well plates in triplicate (100 μL of cell suspension/well) at a density of 1 × 10^4^ cells/well and left for 24 h to attach. Then, culture medium was removed and cells were incubated in 37 °C with 100 μL of working solutions of αM, V, **G3^gh2B5M^**, **G3^2B12gh5M^**, **G3^gh4B5V^** or **G3^2B12gh5V^** for 48 h. After that, NR or XTT assays were performed as earlier described [31].

#### 2.7.3. Proliferation

Cells proliferation was estimated using Hoechst 33,342 staining. BJ, U-118 MG and SCC-15 cells at a density of 5 × 10^3^/well were seeded into 96-well plates and incubated for 24 h at 37 °C to attach. Then, following the culture medium removal, cells were incubated with 100 μL of working solutions of the studied drugs or dendrimer conjugates for 72 h. Afterwards, plates were centrifuged (5 min, 700 g) and the working solutions of studied compounds removed. Cells were fixed in 3.7% formaldehyde solution in PBS for 10 min at room temperature and again centrifuged followed by staining with 1 µg/mL Hoechst 33,342 solution in PBS (100 μL, 1 h). The fluorescence signal was measured using Tecan Infinite M200 PRO Multimode Microplate Reader (TECAN Group Ltd., Männedorf, Switzerland) at 350/461 nm.

#### 2.7.4. Toxicity to *Caenorhabditis elegans* and the Worm Survival Analysis

*Caenorhabditis elegans* nematodes (wild type strain N2, variety Bristol) were cultured at 20 °C on NGM agar plates with *E. coli* OP50 lawn as a food source [40]. Worm survival analysis was performed as described in [29].

#### 2.7.5. Statistical Analysis

In order to estimate the differences between treated cells and non-treated control groups, statistical analysis was performed using the non-parametric Kruskal-Wallis test due to the lack of a normal distribution of the data. To determine the statistically significant differences between the ester conjugate-treated group (**G3^gh2B5M^** or **G3^gh4B5V^**) against the amide conjugate-treated group (**G3^2B12gh5M^** or **G3^2B12gh5V^**), a Mann–Whitney U test was applied (*p* ≤ 0.05 was considered statistically significant). The survival curves of *C. elegans* nematodes were presented in a plot of the Kaplan–Meier estimator. Gehan’s Wilcoxon test was used to assess statistically significant differences (with *p* ≤ 0.05) between treated and non-treated control nematodes. All analyses and calculations were performed with Statistica 13.3 software (StatSoft Poland, Cracow).

## 3. Results and Discussion

### 3.1. Syntheses and Characterization of Dendrimer Conjugates

The PAMAM G3 dendrimer was converted by exhaustive glucoheptoamidation with GHL as described before [39] to obtain **G3^gh^** (Scheme 1A). This 2 nm sized dendrimer was then used as a core to attach αM or V and biotin (B). The αM was modified by ester attachment of succinyl linker (αM-Suc) via 6-O (Scheme 1B) as was determined before [39]. At the step of conjugate formation, the carboxyl groups of αM-Suc or V, and B were activated efficiently with Mukaiyama reagent (2-chloro-1-methylpyridinium iodide) in the presence of 4-(dimethylamino)pyridine (DMAP), enabling further reaction with the **G3^gh^** core hydroxyl groups. Mukaiyama reagent is commonly used for synthesis of esters, lactones, amides and ketenes, causing activation of hydroxyl groups of carboxylic acids and alcohols [41]. The products were isolated by extensive removal of low molecular reagents and side-products by dialysis and were characterized by NMR spectroscopy and DLS. The synthesis pathway and conjugate formula with atom numbering are presented in Scheme 2.

The ^1^H NMR spectrum of **G3^gh^** dendrimer conjugated with αM and biotin via ester bonds is presented in Figure 1B compared to αM alone (Figure 1A) (both measurements were performed in DMSO-d_6_).

Additional 2-D COSY, HSQC and HMBC measurements were performed (Figures S1 and S2) and allowed for detailed assignments of ^1^H and ^13^C resonances (Table S1). The number of attached residues of αM and B to **G3^gh^** dendrimer was estimated based upon the integral intensity of αM aromatic protons (5^M^ at 6.79 ppm and 4^M^ at 6.34 ppm) and one free hydroxyl proton (1′^M^ at 13.73 ppm) resonances, and B resonances (10^B^ and 11^B^ at 6.48 and 6.40 ppm) in relation to the reference intensity of b_0-3_ protons resonance from the dendrimer core, namely [120H] (for detailed assignment of protons from internal PAMAM G3 arms see Figure 2 in [29]). The averaged final product was identified as **G3^gh2B5M^**. In its ^1^H-NMR spectrum, the characteristic resonances of αM protons are observed and retained the same chemical shifts as resonances of protons recorded in αM alone. Proton resonances from 3′^M^ and 6′^M^ hydroxyl groups were not detected, while singlet resonance at 13.73 ppm corresponding to the third hydroxyl group proton (1′^M^) was found. It suggests that either of the 3′^M^ and 6′^M^ protons participate in bonding to **G3^gh^** dendrimer.

The ^1^H NMR spectrum of **G3^gh^** conjugated with vadimezan and biotin is shown in Figure 2B in comparison with vadimezan (Figure 2A) (both in DMSO-d_6_), while detailed NMR spectral assignments were performed by 2-D COSY, HSQC, and HMBC measurements as presented in Figure S3 (COSY) and Figure S4 (combined heteronuclear ^1^H-^13^C HSQC/HMBC) with Table S1.

The stoichiometry of conjugate was determined by a comparison of integral intensity of V aromatic proton resonances (2^V^ and 7^V^) at 7.50–7.15 ppm, and B resonances (10^B^ and 11^B^ at 6.50–6.35 ppm) versus CH_2_(b_0-3_) PAMAM G3 core resonance centered at 2.20 ppm ([120H]); additional intensity comes from 4 biotin CH_2_ multiplet identified in COSY (Figure S3, cross-peak q). The average conjugate was identified as **G3^gh4B5V^**. Only 2^V^ and 7^V^ aromatic protons resonances were recorded as isolated signals, while the remaining signals of vadimezan protons are overlapped by resonances of dendrimer and gh protons. 2-D COSY, HSQC and HMBC measurements allowed for detailed assignment of each signal. It is worth noting that within glucoheptoamide residues the CH resonances were identified unambiguously (in COSY cross-peak j is between 2^gh^ and 3^gh^), while scalar couplings within hydroxymethylene groups HO-C-H were observed (cross-peaks e, f, h, and g in COSY experiment, see Figure S3), which enabled to combine vicinal OH and H in gh residues (Table S1).

PAMAM G3 dendrimer conjugate with amide-bonded vadimezan, biotin, and GHL was synthesized to compare its activity with that of the αM dendrimer conjugate (**G3^2B12gh5M^**) obtained and described earlier [29]. As in the synthesis of the ester conjugates described above, Mukaiyama reagent was also used in the synthesis of the amide conjugate with V. In this procedure, native PAMAM G3 was used as a conjugate core. In the presence of Mukaiyama reagent and DMAP, amine groups from the dendrimer surface were able to react with carboxyl groups of V and B. The intermediate product, isolated by dialysis and characterized by NMR spectroscopy, was identified as G3^2B5V^ and further converted into half-glucoheptoamidated derivative by the substitution of half of the remaining amine groups with GHL. The synthesis pathway and conjugate formula with atom numbering are presented in Scheme 3. The ^1^H NMR spectrum of the conjugate in DMSO-d_6_ is shown in Figure 2C. The stoichiometry of the conjugate was estimated in the same way as described above for ester-bonded vadimezan conjugate and allowed us to identify product as **G3^2B12gh5V^**.

The conjugates were obtained in rather low yield (<55%) due to compromising it against the high purity reached by extensive dialysis.

### 3.2. Size and ζ Potential of Dendrimer Conjugates

DLS measurements were performed to determine the size and zeta potential of the synthesized conjugates. Obtained values are collected in Table 1 and illustrated in Figures S5 and S6.

The dynamic diameter of **G3^gh^** in an aqueous solution is 1.5 nm (averaged by number) or 1.7 nm (averaged by volume), which is nearly two-fold smaller than diameter of the native PAMAM G3. In the acidic environment (pH 5) the diameter of **G3^gh^** increases to 3.6 nm (d(N)) or 4.3 nm (d(V)) upon protonation of internal tertiary amine groups, which was observed earlier for the series of G3 substituted with variable amount of gh residues [42]. Slightly higher dynamic diameter of **G3^gh4B5V^** conjugate, equal 2.9 nm (by number) or 3.7 nm (by volume) in water and 4.2 nm (d(N)) or 4.9 nm (d(V)) in pH 5, indicates that this conjugate is dispersed at unimolecular level. The other conjugates were associated both in pH 7 and pH 5, with the strongest effect observed for **G3^2B12gh5M^** and **G3^2B12gh5V^**. The molecules of the ester conjugate **G3^gh2B5M^** also associate but particle size is 10 times smaller than that of the amide **G3^2B12gh5M^** analogue. However, the particle size of **G3^2B12gh5M^** was reduced by seven times upon a pH decrease from 7 to 5. This effect can be attributed to the presence of ca. 12 free amine groups in the **G3^2B12gh5M^** conjugate which are readily to protonate, while amine groups in ester conjugate are fully glucoheptoamidated.

All studied conjugates have positive zeta potential. For conjugates with small molecular size, **G3^gh^** and **G3^gh4B5V^** zeta potential values are <10 mV and increase with the conjugates diameter up to 33.4 mV and 37.5 mV for **G3^2B12gh5V^** and **G3^2B12gh5M^**, respectively. It is also worth noting that amide conjugates, **G3^2B12gh5M^** and **G3^2B12gh5V^** have comparable zeta potential and particle size but they differ in biological activity (vide infra). Due to the fact that the cell membrane is negatively charged, nanoparticles with positive net charge have a greater ability to enter and penetrate cells [43].

### 3.3. Cytotoxicity

The NR and XTT assays revealed that vadimezan (V) after 48 h incubation showed no cytotoxic effect against any studied cell line (BJ, SCC-15 and U-118 MG) up to 200 µM concentration (Table 2). Vadimezan is a Vascular Disrupting Agent (VDA) whose anticancer action, based mainly on an irreversible destruction of established tumor vessels and tumor blood flow arrest, has been observed mainly in the endothelial cells where it induced apoptosis [14]. However, the present results do not indicate V to lower viability of cells, including epithelial cancer cells (SCC-15). Our results are consistent with other, pointing to the drug’s selective toxicity against vascular endothelial cells. Using the MTT assay, Zhang et al. [44] showed that V did not disturb the mitochondrial metabolism of any of ten cell lines (Ishikawa, A549, Bewo, HeLa, Siha, MCF-7, HL-60, BEL-7402, NCI-460, BGC-823) after 48 h incubation. Lv et al. [45] also found free V (up to 35.4 μM) to be non-toxic to breast carcinoma MCF-7 cells, lung carcinoma A549 cells and mouse embryo fibroblast NIH/3T3 cells after 48 h treatment. Only human vein umbilical cell (HUVEC) growth was demonstrated to undergo inhibition by vadimezan (IC_50_ > 20 µM) [44]. On the other hand, the delivery of nanoparticles (mPEG-*b*-PHEA) conjugated with V and encapsulated with DOX resulted in cytotoxicity to both tumorous and nontumorous cells. Furthermore, those nanoparticles equipped with V and DOX showed a similar inhibition of cells proliferation to free DOX [45].

The anticancer activity of xanthones depends on the type, number, and position of the attached functional groups in their skeleton [47]. Anticancer activity of αM seems to depend on the presence of two isoprenyl groups localized on 2-nd and 8-th carbon in xanthenone ring [29], not present in vadimezan. Castanheiro et al. [48] showed that the introduction of the prenyl group to the 1-hydroxyxanthone markedly increased its anticancer activity against the MCF-7 cell line, in accordance with the presence of prenyl groups being associated with an improvement of potency and selectivity of xanthones [49]. At present, prenylated xanthones are the most valued xanthones, in view of their promising properties as active and selective anticancer agents [47].

**Figure 3:**
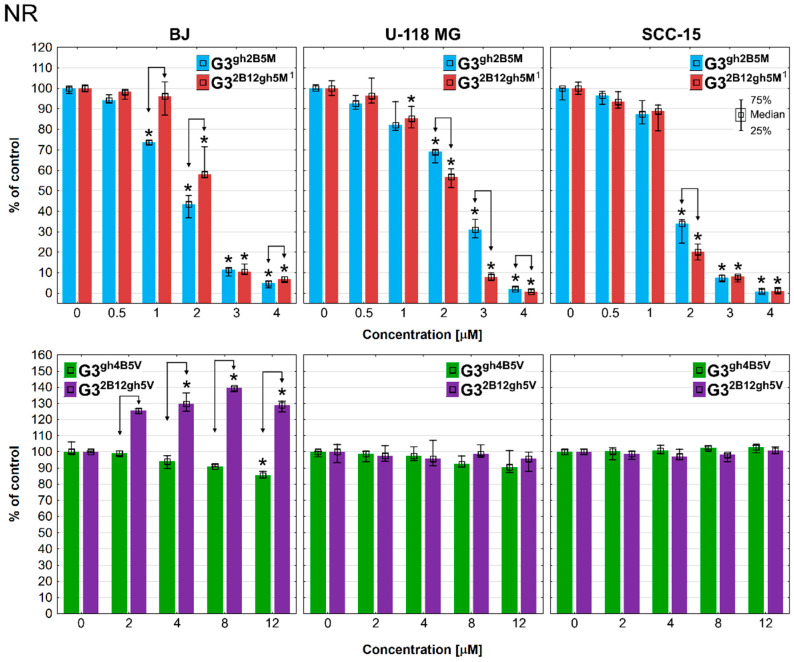
Cytotoxic effect of dendrimers conjugated via ester or amide bond with αM (**G3^gh2B5M^** and **G3^2B12gh5M^**) and V (**G3^gh4B5V^** and **G3^2B12gh5V^**) after 48 h of incubation with normal fibroblasts (BJ), glioblastoma cells (U-118 MG), and squamous carcinoma cells (SCC-15), estimated with the NR assay. Cell viability (expressed as percent of the non-treated control) medians are plotted. The whiskers are lower (25%) and upper (75%) quartile ranges. * *p* ≤ 0.05; Kruskal-Wallis test (against non-treated control), ↕ *p* ≤ 0.05; Mann-Whitney U test (ester against amide-attached drug-conjugate). ^1^ Data for **G3^2B12gh5M^** were reproduced from [29].

**Figure 4:**
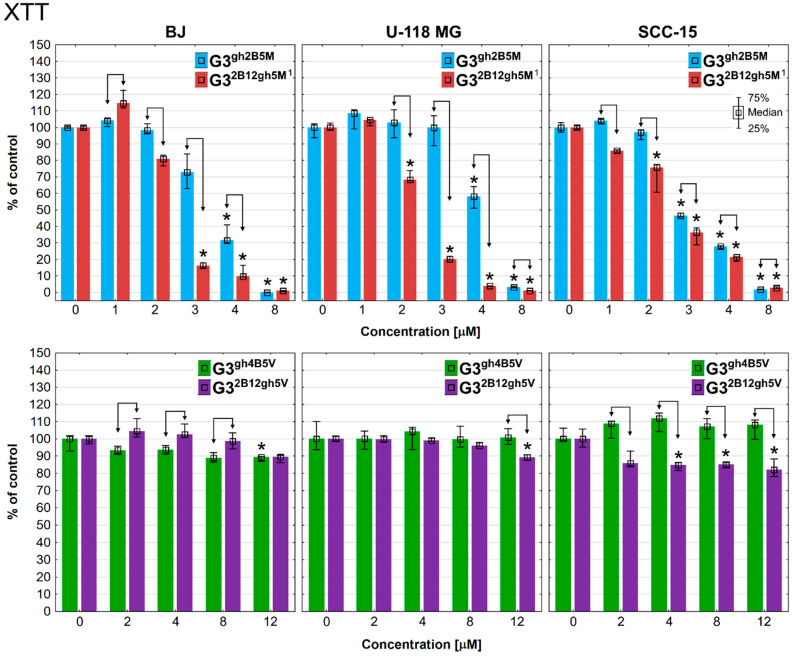
Viability of normal fibroblasts (BJ), glioblastoma cells (U-118 MG) and squamous carcinoma cells (SCC-15) after 48 h of incubation with **G3^gh2B5M^**, **G3^2B12gh5M^**, **G3^gh4B5V^** and **G3^2B12gh5V^**, assessed by XTT assay. Cells viability (expressed as percent of the non-treated control) medians are plotted. The whiskers are lower (25%) and upper (75%) quartile ranges. * *p* ≤ 0.05; Kruskal-Wallis test (against non-treated control), ↕ *p* ≤ 0.05; Mann-Whitney U test (ester- against amide-attached drug-conjugate). ^1^ Data for **G3^2B12gh5M^** were reproduced from [29].

The present results reflect an attempt to increase the anti-tumor activity of V and αM by covalent binding the drugs to biotinylated PAMAM G3 dendrimers, demonstrated previously to be effective in this respect [50]. Cytotoxicity assays revealed that vadimezan attached to biotinylated and glucoheptoamidated PAMAMs did not exert any significant inhibitory activity (up to 24 µM **G3^gh4B5V^** or 12 µM **G3^2B12gh5V^** after 48 h incubation (Table 2, Figure 3 and Figure 4). It should be added that the latter observation provided additional evidence that the applied V vehicles do not show biological activity (*cf*. Figure S7 for **G3^gh^** and [29] for **G3^2B12gh^**. Of note is (Figure 3 and Figure 4) that 4–12 µM **G3^2B12gh5V^** lowered SCC-15 cell viability down to 80% (apparent only in the XTT assay) and 2–12 µM **G3^gh4B5V^** increased BJ cell viability by 25–40% (apparent only in the NR assay). This effect might be caused by changes in cell proliferation rate, as discussed below.

The activity of αM increased significantly after attaching it to the studied carriers. The action of conjugate **G3^2B12gh5M^** was slightly stronger than **G3^gh2B5M^** with an IC_50_ of approx. 1.4–2 µM (NR assay) and 2–2.5 µM (XTT assay) for all studied cell lines. The largest differences were observed in glioma cells, where **G3^2B12gh5M^** acted two-fold stronger than **G3^gh2B5M^**. Moreover, the strongest effect of both conjugates was still observed in SCC-15 cells (Table 2, Figure 3 and Figure 4).

The greater effect of an αM-amide conjugated dendrimer may result from the fact that it has free amino groups responsible for the cytotoxic effect. As it was presented in Section 3.2. the zeta potential of the **G3^2B12gh5M^** conjugate was higher than that of **G3^gh2B5M^**. It could be the reason of a higher activity of the former, since dendrimers with higher zeta potential and surface charge indicate higher cytotoxicity also in consequence of more efficient cellular uptake [51,52]. An important issue is the mechanism of possible enzymatic degradation of amide or ester bonds in conjugates with the participation of lysosomal or other intracellular enzymes and the consequent release of attached drugs. Kurtoglu et al. have shown that PAMAM dendrimer–ibuprofen conjugates with ester or amide linkers differ in the degree of drug release. Amide and ester-linked conjugates were very stable after 48 h of incubation in buffer. The ester-linked conjugates showed pH-dependent release and the extent of release varied with pH from 3% (pH 5) to 38% (pH 8.5) within a 10-day studied period. Moreover, direct amide- and ester-linked conjugates did not release ibuprofen enzymatically in cathepsin B buffer and diluted human plasma [28]. This means that the incubation time of both tested dendrimers (**G3^2B12gh5M^** and **G3^gh2B5M^**) with cells in FBS containing medium was too short to observe significant differences in the release of αM from the conjugates. This was also confirmed by the results of our previous studies concerning the release of doxorubicin conjugated through amide linker to glucoheptoamidated PAMAM G3 dendrimer [39]. Although ester bonds are generally easy to hydrolyze (such as carboxylate ester bond), some ester structures, such as alkyl esters, hydrolyze very slowly, even in the presence of esterase [27]. This could be the reason of weaker activity of dendrimeric conjugates with ester-bonded αM. Quintana et al. [53] and Thomas et al. [54] used, as a model drug, methotrexate (MTX), having two available functional groups for conjugation, carboxylic and amine, in order to examine the effect of the type of chemical bond on the conjugate activity. MTX conjugated to acetamide-functionalized G5 PAMAM dendrimer through ester bond was found four times more active than free MTX, whereas MTX conjugated via amide bonds to the same dendrimer was less active compared to free MTX. However, in this case, changing the binding site of the drug molecule may induce changes in its activity.

An important factor influencing the toxicity of nanoparticles is the efficiency and the pathway they enter cells that depends on their size and charge. All studied conjugates indicated positive charge on their surface. The smallest conjugates (**G3^gh^** and **G3^gh4B5V^**) can probably enter cells through passive uptake, while slightly larger compound **G3^gh2B5M^** (ca. 113 nm diameter) via clathrin-mediated endocytosis. In the case of **G3^2B12gh5M^** and **G3^2B12gh5V^** conjugates, the most possible route of internalization seems to be phagocytosis and/or micropinocytosis (associates diameter ca. 1000–1300 nm) [55]. This phenomenon requires further research.

### 3.4. Proliferation

**Figure 5:**
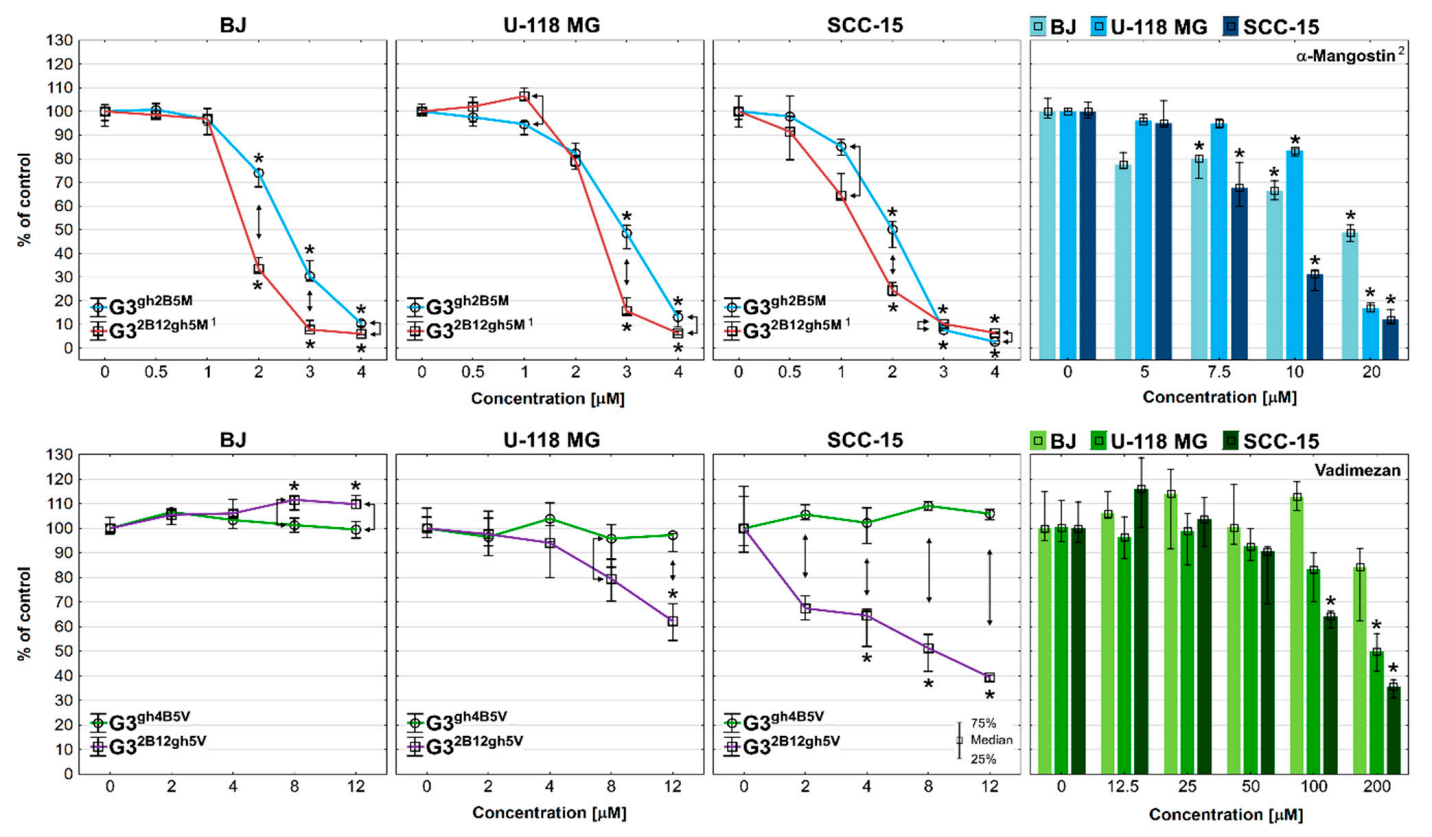
Anti-proliferative effect of **G3^gh2B5M^**, **G3^2B12gh5M^**, **G3^gh4B5V^**, **G3^2B12gh5V^**, αM, and V on BJ, U-118 MG, and SCC-15 cells after 72 h of incubation, determined with Hoechst 33,342 staining. Medians of the fluorescence signal (expressed as percent of the non-treated control), indicating the cell number in a well, are plotted. The whiskers indicate lower (25%) and upper (75%) quartile ranges. * *p* ≤ 0.05; Kruskal-Wallis test (against non-treated control), ↕ *p* ≤ 0.05; Mann-Whitney U test (ester against amide-attached drug-conjugate). ^1,2^ Data for **G3^2B12gh5M^** conjugate and αM were reproduced from [2,29], respectively.

There are no clear examples of the antiproliferative activity of vadimezan in the available literature, which suggests that such properties were not observed at therapeutic concentrations. One paper showed that the combination of tCoa-NGR fusion proteins with V suppressed the growth and proliferation of B16-F10 melanoma tumors in C57/BL6 mice [56]. In our studies, V inhibited the proliferation of cancer SCC-15 cells from 100 µM concentration and U-118 MG glioma cells from 200 µM, but not normal cells (Figure 5). PAMAM G3 dendrimer, biotinylated and partially glucoheptoamidated with V bound via amide linker (**G3^2B12gh5V^**), enhanced its anti-proliferative activity by as much as 50-fold. This construct distinctly limited proliferation of SCC-15 and U-118 MG cells when applied at concentrations 2 and 12 µM, respectively. This effect apparently was related to the decrease of mitochondrial efficiency observed at 4–12 μM **G3^2B12gh5V^** in SCC-15 and U-118 MG cells(Figure 4). Of note is that in normal BJ cells **G3^2B12gh5V^** rather promoted cell proliferation at 8 and 12 µM concentrations (Figure 5), suggesting **G3^2B12gh^** vehicle to be responsible for this effect, as it significantly increased fibroblasts growth at the 5–20 μM range of concentrations [29]. No effect was observed with the conjugate containing the ester linkage (**G3^gh4B5V^**), despite the fact that the 20 µM **G3^gh^** vehicle itself showed a weak anti-proliferative effect against SCC-15 cells (Figure S7, part C). The size and zeta potential of the studied conjugates may be important factors influencing their properties, as the **G3^2B12gh5V^**, compared to **G3^gh4B5V^** conjugate, showed higher activity and formed associations with diameter at pH 7 being about 300-fold larger (about 1000–1200 nm vs. 2.90–3.7 nm). Thus, it may increase the polyvalence of conjugates, which is responsible for their increased activity [57]. The size of the molecule and the presence of free amino groups on the **G3^2B12gh5V^** surface corresponded to its 3-fold higher zeta potential, responsible for a stronger toxicity of dendrimers [51]. However, it seems that this was a rather minor effect, since this phenomenon was not seen in normal BJ fibroblasts (BJ cells are more sensitive to high surface charge of dendrimers than SCC-15 cancer cells [58]).

Unlike vadimezan, αM is a well-known anti-proliferative agent. The inhibition of cell proliferation induced by αM led to G1-phase arrest and S-phase suppression in MDA-MB231 human breast cancer cells with altering the expression of cell-cycle-related molecules, such as p21^cip1^, CHEK2, cyclins, CDKs, and PCNA [59]. The G0/G1 phase arrest and the G1 arrest caused by αM was also observed in four hepatocellular carcinoma (HCC) cell lines [9] and human oral squamous carcinoma cells (OSCC) [60], respectively. In our study, the proliferation of cancer cells (U-118 MG and SCC-15) and normal BJ fibroblasts was studied after 72 h of incubation with each of the xanthones (αM and V) and their dendrimer conjugates. The results are presented in Figure 5 and demonstrate anti-proliferative activity of αM in the concentration range of 7.5–20 μM [2]. The strongest inhibition of proliferation was observed for SCC-15 cells (reduction to 12% at 20 μM), slightly weaker for glioma cells (reduction to 17% at 20 μM) and the weakest for normal fibroblasts (reduction to 49% at 20 μM).

Conjugation of biotinylated dendrimers with 5 residues of αM enhanced its anti-proliferative activity against both cancer and normal cell lines, with the **G3^gh2B5M^** ester conjugate acting slightly weaker (over 3-fold enhancement) than the **G3^2B12gh5M^** amide conjugate (over 5-fold enhancement). The proliferation of SCC-15 cells underwent the strongest inhibition by these two αM conjugates (almost no viable cells at 3 and 4 μM, respectively). The latter effect was less pronounced in the case of normal cells (30% viable cells at 3 μM **G3^gh2B5M^** and 34% viable cells at 2 μM **G3^2B12gh5M^**), with glioblastoma cells being the least sensitive (49% and 16% at 3 μM **G3^gh2B5M^** and **G3^2B12gh5M^**, respectively). The most significant difference in suppression of cell growth elicited by αM ester or amide conjugates was observed with fibroblasts (74% and 33% viable cells at 2 μM **G3^gh2B5M^** and **G3^2B12gh5M^**, respectively).

### 3.5. Toxicity against C. elegans and the Effect on the Worm Survival

Biological activities of αM and V, used as free compounds or their dendrimer conjugates, were also evaluated with a model nematode, *Caenorhabditis elegans*, a cost-effective and powerful model widely used for screening of bioactive compounds prior to be tested with higher animals [37]. *C. elegans* has been previously used in studies on the toxicity of drug-loaded nanoparticles, such as glibenclamide-loaded PVM/MA (poly(methylvinyl ether-co-maleic anhydride)) nanoparticles for oral delivery purposes [61], insulin-loaded PPA (PEG-polu(anhydride) conjugate) nanoparticles with the ability to mucus permeation for the oral administration of insulin [62], and the toxicity of bare nanoparticles, for example, zinc oxide nanoparticles (ZnO-NPs) [63] and gold nanoparticles (AuNPs) [64].

**Figure 6:**
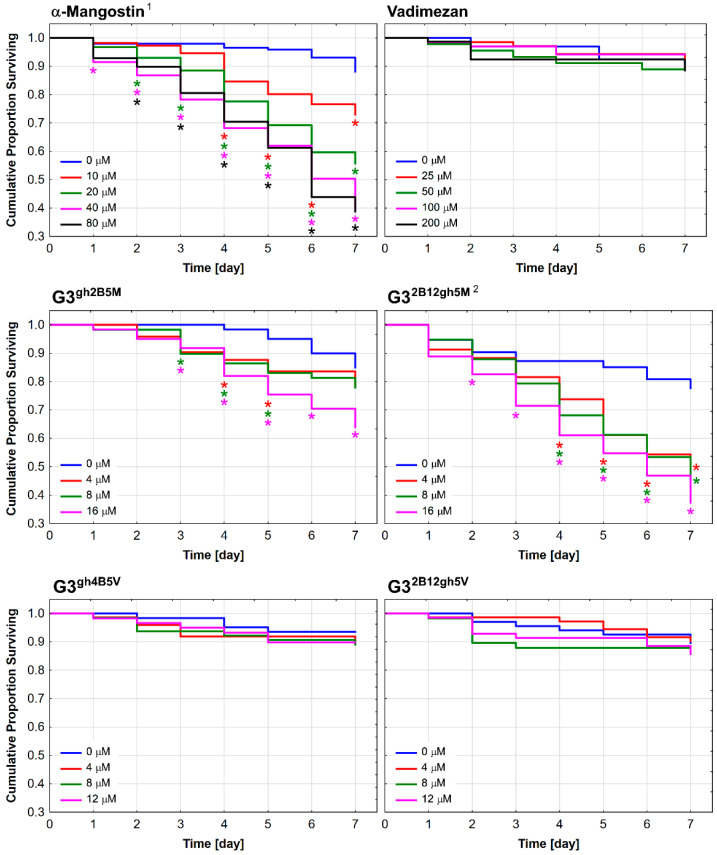
The Kaplan-Meier survival curves of *C. elegans* after 7 days of incubation with αM, V, **G3^gh2B5M^**, **G3^2B12gh5M^**, **G3^gh4B5V^** or **G3^2B12gh5V^**. Results are presented as cumulative proportion surviving. Statistically significant differences against dmso-treated control obtained in Gehan’s Wilcoxon test are marked with asterisks * (*p* ≤ 0.05) in the colours corresponding to the tested concentrations. ^1,2^ Data for αM and **G3^2B12gh5M^** conjugate were reproduced from [2,29], respectively.

We used synchronized population of L4-stage nematodes to examine survivability and general toxicity after 7 days of incubation with free αM or V, and with their dendrimer conjugates: **G3^gh2B5M^**, **G3^2B12gh5M^**, **G3^gh4B5V^**, **G3^2B12gh5V^** at different concentration ranges. The results of worms’ survival are shown in Figure 6 and in Table 3 (LC_50_ values).

Free V exhibited no toxicity against *C. elegans* up to 200 μM concentration with a cumulative proportion surviving equal to 90%. The conjugation of V to the dendrimer by either ester or amide bonds also did not increase its toxicity. On the other hand, αM caused a toxic effect with the LC_50_ value, the minimum being over 10 times higher than that of V (comparing LC_50_ values). This confirms that the presence of prenyl residues in αM structure has a significant influence on its toxicity in vivo. Substitution of αM with biotinylated and glucoheptoamidated dendrimers via ester linker (**G3^gh2B5M^**) increased its toxicity only by 20%, while conjugate with amide-bonded αM (**G3^2B12gh5M^**) was more active by 140% comparing to free αM (Table 3). Additionally, **G3^gh2B5M^** conjugate showed two-fold weaker activity than **G3^2B12gh5M^** in the worm.

## 4. Conclusions

Similar toxicity profiles of the tested conjugates in vitro and in vivo allow us to conclude that the main factors influencing the differences in the activity of evaluated agents were: differences in the value of the zeta potential and the size of the nanoparticles, as well as the presence of various linkers (amide or ester). The results clearly show that conjugates containing amide bonds, partially-blocked amino groups on the surface, larger particle diameters and higher zeta potentials could be a more effective tool for therapy for cancer and nematode infections. Considering the vadimezan mechanism of action, based on its selective effect on blood vessel cells in the tumor environment, conjugates with vadimezan are still valuable research objects. An antiproliferative effect of **G3^2B12gh5V^** in vitro against cancer, but not normal cells, is also interesting. Therefore, the proposed drug delivery system based on the PAMAM G3 dendrimer with partially-blocked amine groups on the surface can be a useful tool to improve the biological properties of transported drug molecules. Further studies on the in vivo model with implanted and perfused tumors are necessary.

## Figures and Tables

**Table 1:** Size of conjugates averaged by volume (d(V)) and by number of molecules (d(N)) and zeta potential values ± standard deviation determined by DLS analysis.

Compound	Size [nm]				Zeta Potential [mV]	
	pH 7		pH 5		pH 7	pH 5
	d(V)	d(N)	d(V)	d(N)		
**G3^gh^**	1.7 ± 0.44	1.5 ± 0.51	4.3 ± 0.34	3.6 ± 0.32	4.5 ± 0.58	5.3 ± 0.81
**G3^gh2B5M^**	155.1 ± 3.46	113 ± 5.89	212.7 ± 4.29	144.5 ± 6.49	10 ± 0.39	24.9 ± 1.78
**G3^2B12gh5M^** [29]	1367 ± 245.7	1262 ± 196.4	178.3 ± 5.73	113.8 ± 7.34	22.7 ± 1.01	37.5 ± 2.32
**G3^gh4B5V^**	3.7 ± 0.23	2.9 ± 0.56	4.9 ± 0.14	4.2 ± 0.18	7.6 ± 4.35	6.2 ± 0.81
**G3^2B12gh5V^**	1166 ± 153	970.4 ± 81.6	1090 ± 39.2	924.4 ± 39	22.8 ± 0.69	33.4 ± 2.82

**Table 2:** The half maximal inhibitory concentration (IC_50_) values estimated with NR and XTT assay after 48 h incubation of BJ, U-118 MG and SCC-15 cells with αM or V, each used either as a free drug or the dendrimer conjugate. The IC_50_ values were calculated with AAT Bioquest IC_50_ calculator [46].

**IC_50_ [μM] NR Assay**			
	**BJ**	**U-118 MG**	**SCC-15**
**α-mangostin** [2]	8.97	9.59	6.43
**G3^gh2B5M^**	2.02	2.52	1.7
**G3^2B12gh5M^** [29]	2	1.83	1.41
**Vadimezan**	>>200	>>200	>>200
**G3^gh4B5V^**	>>24	>>24	>>24
**G3^2B12gh5V^**	>>12	>>12	>>12
**IC_50_ [μM] XTT Assay**			
	**BJ**	**U-118 MG**	**SCC-15**
**α-mangostin** [2]	18.58	18.15	7.72
**G3^gh2B5M^**	3.36	4.14	3.02
**G3^2B12gh5M^** [29]	2.37	2.05	2.52
**Vadimezan**	>>200	>>200	>200
**G3^gh4B5V^**	>>24	>>24	>>24
**G3^2B12gh5V^**	>>12	>>12	>>12

**Table 3:** The half maximal lethal concentration (LC_50_) values estimated after 7 days incubation of *C. elegans* nematodes with free αM and V, or their dendrimer conjugates. Data are presented as medians with the first and third quartile. Presented values were calculated with AAT Bioquest IC_50_ calculator [46].

	LC_50_ [μM]	1st Quartile	3rd Quartile
**α-Mangostin** [2]	18.74	18.64	20.23
**G3^gh2B5M^**	>16	>16	>16
**G3^2B12gh5M^** [29]	7.87	4.95	8.09
**Vadimezan**	>>200	>>200	>>200
**G3^gh4B5V^**	>>24	>>24	>>24’
**G3^2B12gh5V^**	>>12	>>12	>>12

## Data Availability

Data supporting the reported results are available on request from the corresponding author.

## Supplementary Materials

The following supporting information can be downloaded at: https://www.mdpi.com/article/10.3390/pharmaceutics14030606/s1, Figure S1: The ^1^H-^1^H COSY spectrum of **G3^gh2B5M^** in DMSO-d_6_; Figure S2: The HSQC/HMBC combined spectra for **G3^gh2B5M^** in DMSO-d_6_; Figure S3: The ^1^H-^1^H COSY spectrum of **G3^gh4B5V^** in DMSO-d_6_; Figure S4: The HSQC/HMBC combined spectra for **G3^gh4B5V^** in DMSO-d_6_; Figure S5: Diameter of conjugates averaged by volume (d(V)) and by number of molecules (d(N)) measured in water (A) and in acetate buffer pH 5 (B); Figure S6: Zeta potential of conjugates measured in water (pH 7) and acetate buffer (pH 5); Figure S7: Cytotoxicity of **G3^gh^** vehicle estimated with NR (A) and XTT (B) assay after 48 h incubation with normal fibroblasts (BJ), glioblastoma cells (U-118 MG) and squamous carcinoma cells (SCC-15); Table S1: The ^1^H and ^13^C resonance assignments of the **G3^gh4B5V^**, V, **G3^gh2B5M^** and αM based on COSY and HSQC/HMBC spectra in DMSO-d_6_.
