# Supplementary material for: Synthesis and Properties of α-Mangostin and Vadimezan Conjugates with Glucoheptoamidated and Biotinylated 3rd Generation Poly(amidoamine) Dendrimer, and Conjugation Effect on Their Anticancer and Anti-Nematode Activities
Source: Pharmaceutics. 2022 Mar 10;14(3):606. doi: 10.3390/pharmaceutics14030606 (PMC8951109; doi:10.3390/pharmaceutics14030606)
Supplement: Supplementary file 1 [file pharmaceutics-14-00606-s001.zip › pharmaceutics-1609770-supplementary.pdf]

pharmaceutics

Supplementary materials: Synthesis and Properties of α-Mangostin and Vadimezan Conjugates with Glucoheptoamidated and Biotinylated 3rd Generation Poly(amidoamine) Dendrimer, and Conjugation Effect on Their Anticancer and Anti-Nematode Activities

Joanna Markowicz, Stanisław Wołowiec, Wojciech Rode and Łukasz Uram

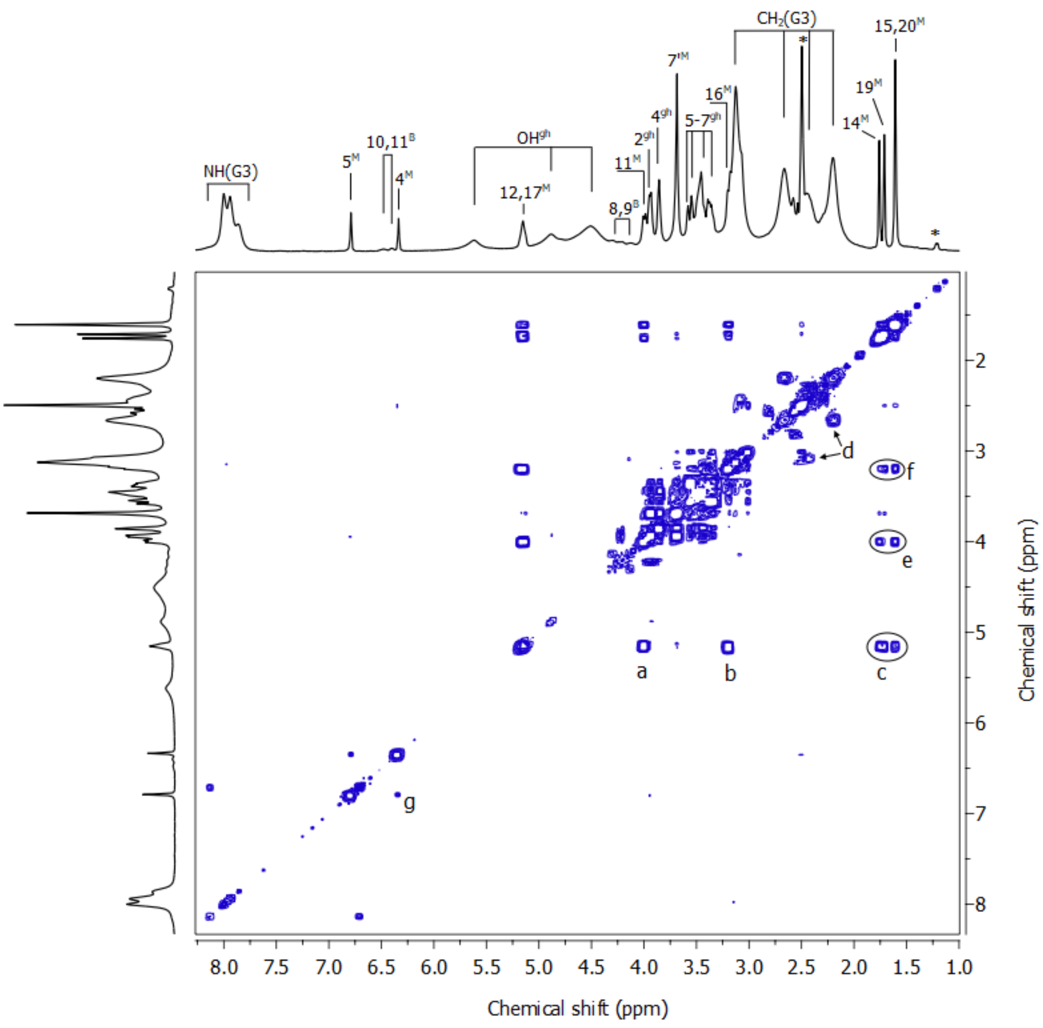

Figure S1. The ^{1}H-^{1}H COSY spectrum of G3^{gh2B5M} in DMSO-d$_6$. The relevant cross-peaks are labeled as follows: a – 11H^M/12H^M; b – 16H^M/17H^M; c – a group of scalar coupling peaks between 14H^M, 15H^M, 19H^M, 20H^M (methyl group singlets) and 12H^M, 17H^M (overlapping methylene triplets); d – a group of internal PAMAM G3 cross-peaks; e – 14H^M,15H^M/11H^M; f – 19H^M,20H^M/16H^M; g – 4H^M/5H^M. The chemical shifts of ^{1}H peaks are collected in Table S1.

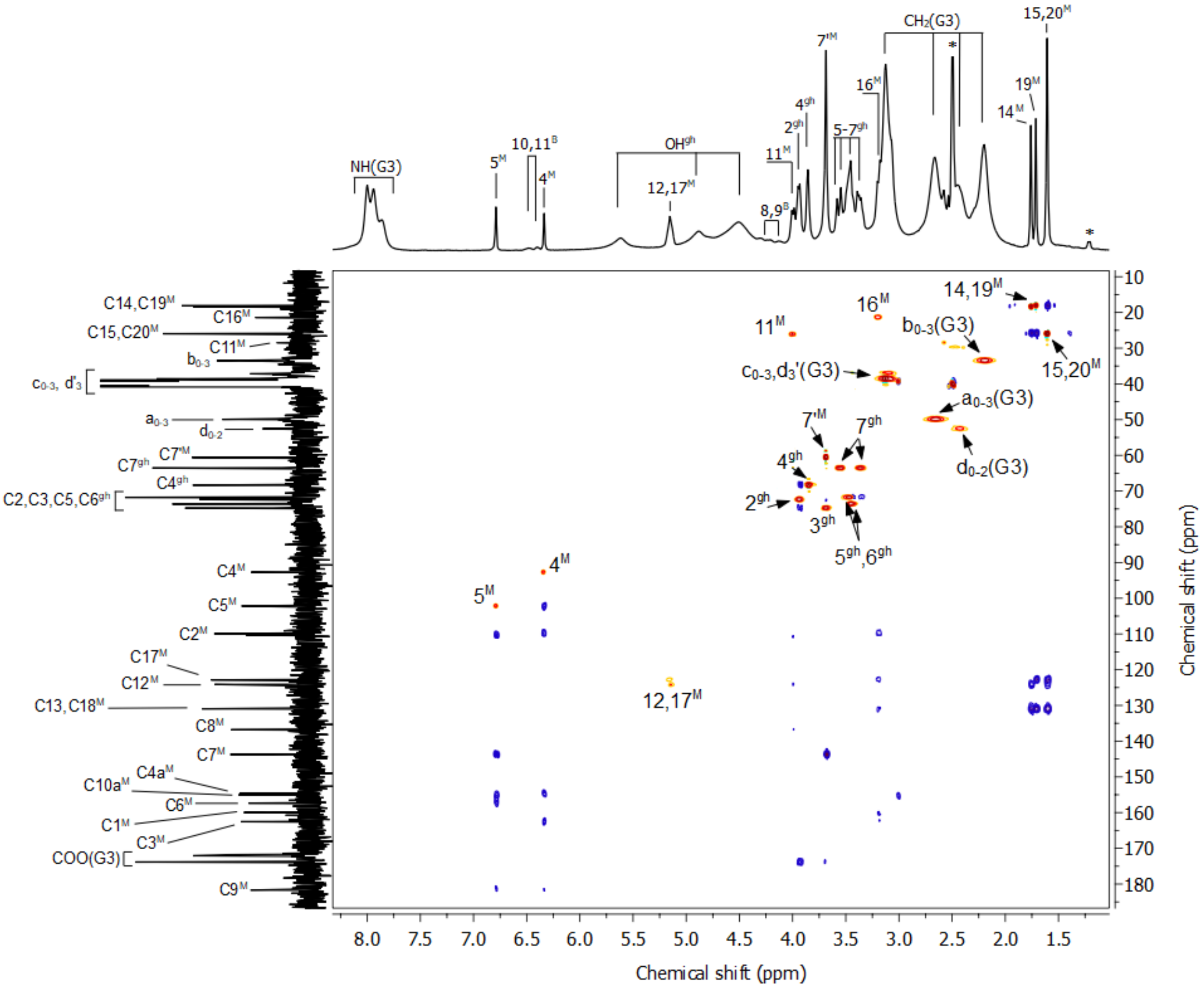

Figure S2. The HSQC/HMBC combined spectra for G3^{gh2B5M} in DMSO-d$_6$. HSQC peaks are marked yellow-red and HMBC peaks are marked blue. The one-bond ^{1}H-^{13}C peaks are labeled with locant and substituents with upper case (^{M}, ^{B}, and ^{gh}), while the peaks within PAMAM G3 arms are labeled according to numbering shown in Fig.2 in [29]. The chemical shifts of ^{1}H and ^{13}C peaks are collected in Table S1.

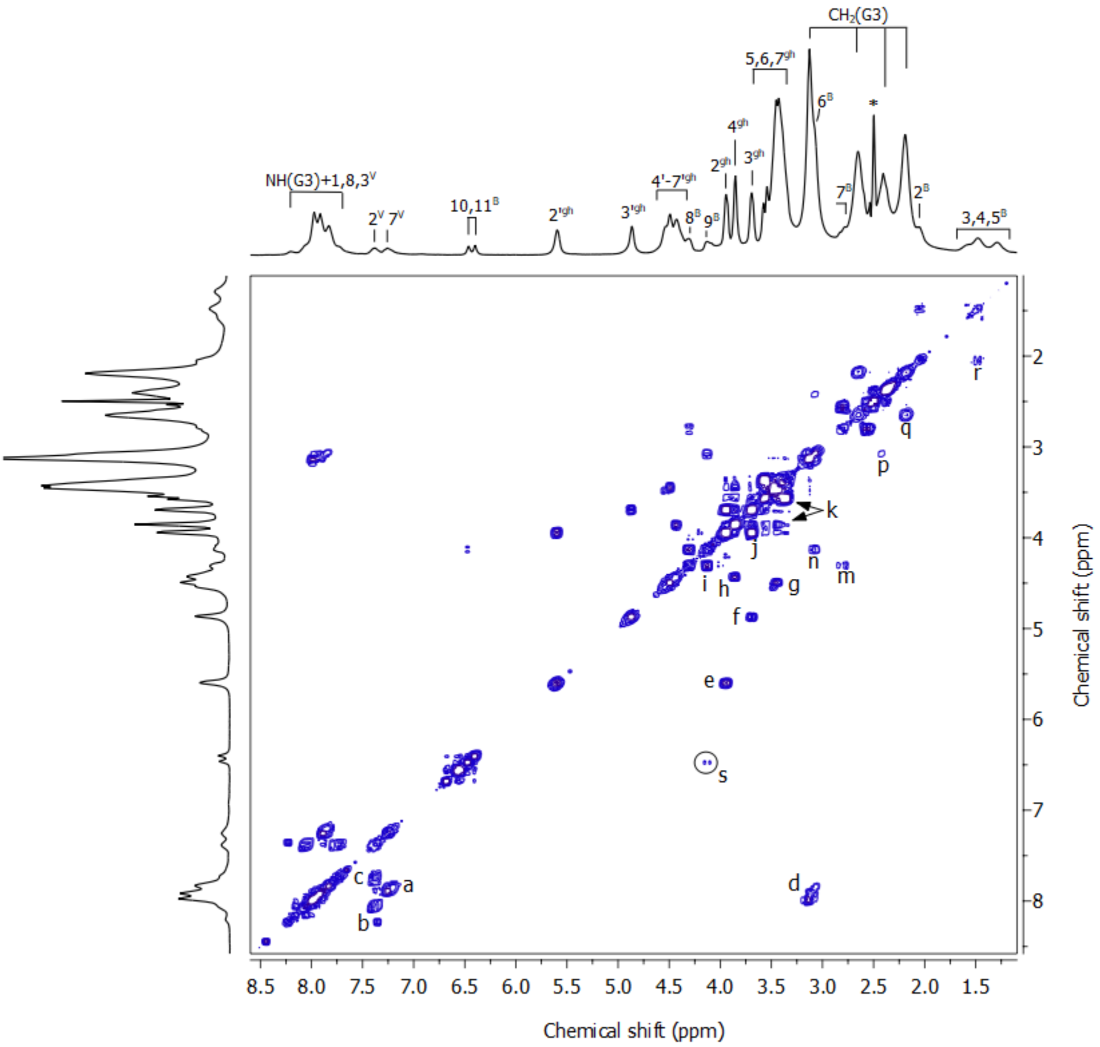

Figure S3. The ^{1}H-^{1}H COSY spectrum of $G3^{gh4B5V}$ in DMSO-d_6. The relevant cross-peaks are labeled as follows: a – $7H^{V}/8H^{V}$; b – $2H^{V}/1H^{V}$; c – $2H^{V}/3H^{V}$; d – $c_{0\text{-}3}$/NH (internal PAMAM G3 cross-peak; for detailed assignment see Fig. 2 in [29]); e – $2H^{gh}/2'H^{gh}$; f – $3H^{gh}/3'H^{gh}$; g – cross-peak between group of gh CH protons (5^{gh}, 6^{gh}, 7^{gh}) and group of OH protons ($5'^{gh}$, $6'^{gh}$, $7'^{gh}$) in gh; h – $4H^{gh}/4'H^{gh}$; i – $9H^{B}/8H^{B}$; j – $3H^{gh}/2H^{gh}$,$4H^{gh}$; k – $5H^{gh}/4H^{gh}$; m – $7H^{B}/8H^{B}$; n – $6H^{B}/9H^{B}$; p – $d_{0\text{-}2}/c_{0\text{-}3}$ and q – $b_{0\text{-}3}/a_{0\text{-}3}$ (internal PAMAM G3 cross-peaks); r – $3H^{B}/2H^{B}$; s – $9H^{B}/11H^{B}$. The chemical shifts of ^{1}H peaks are collected in Table S1.

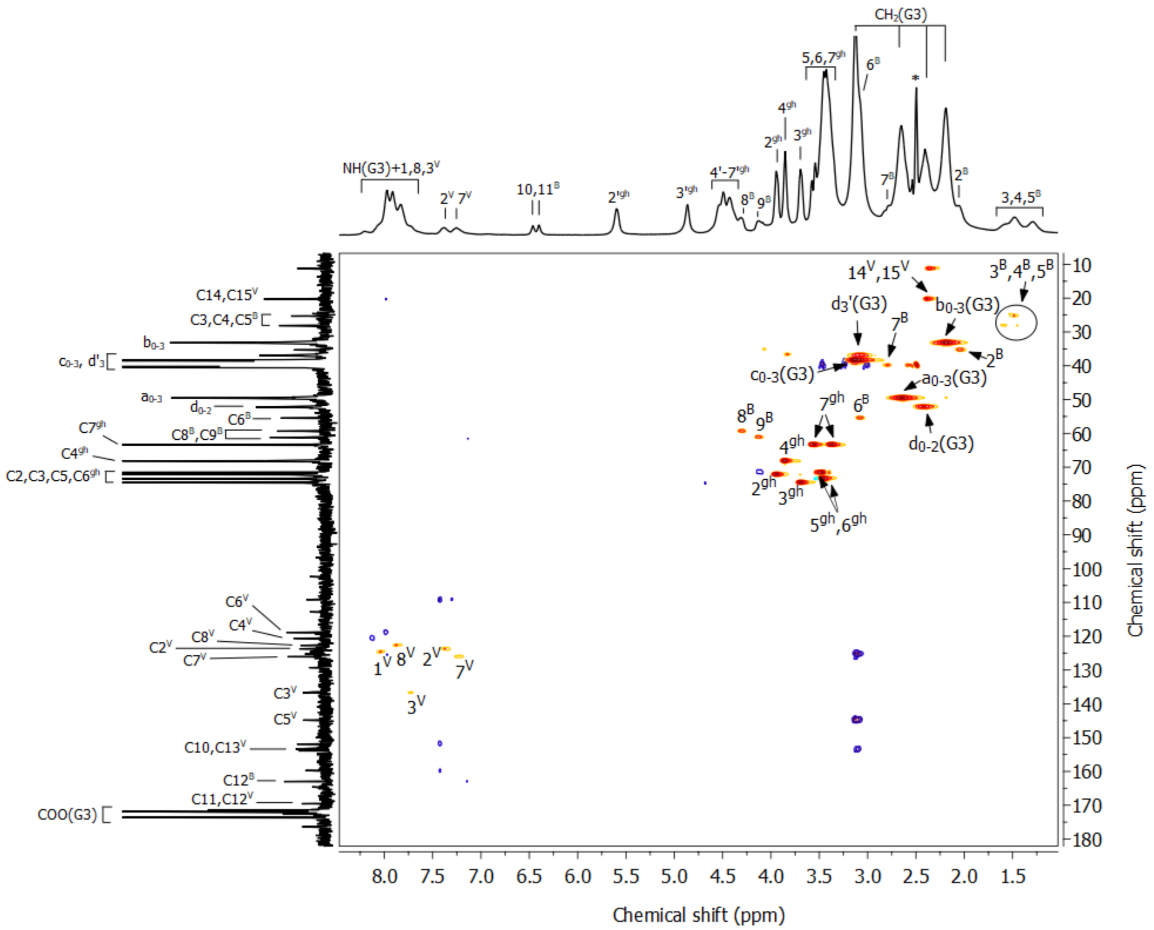

Figure S4. The HSQC/HMBC combined spectra for G3^{gh4B5V} in DMSO-d$_6$. HSQC peaks are marked yellow-red and HMBC peaks are marked blue. The one-bond ^{1}H-^{13}C peaks are labeled with locant and substituents with upper case (^{V}, ^{B}, and ^{gh}), while the peaks within PAMAM G3 arms are labeled according to numbering shown in Fig.2 in [29]. The group of HSQC peaks between 3, 4, and 5 proton nuclei of biotin and corresponding carbon nuclei are grouped within the circle on the map. The chemical shifts of ^{1}H and ^{13}C peaks are collected in Table S1.

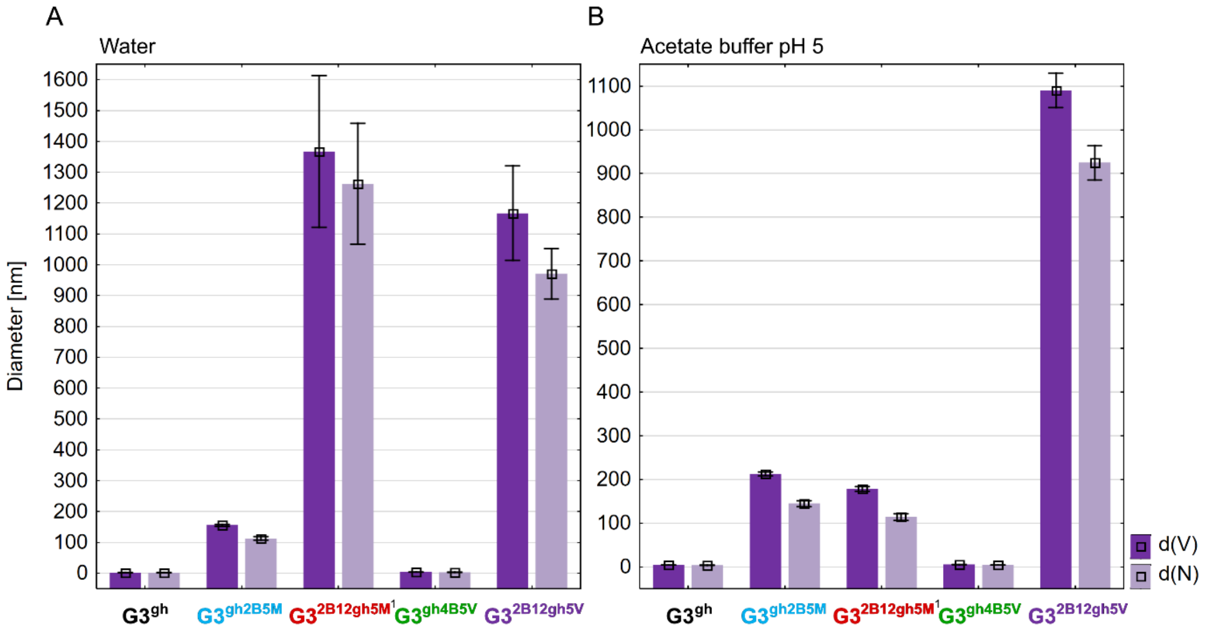

Figure S5. Diameter of conjugates averaged by volume (d(V)) and by number of molecules (d(N)) measured in water (A) and in acetate buffer pH 5 (B). Data are presented as a mean ± standard deviation. [1] Values for G3^{2B12gh5M} were reproduced from [29].

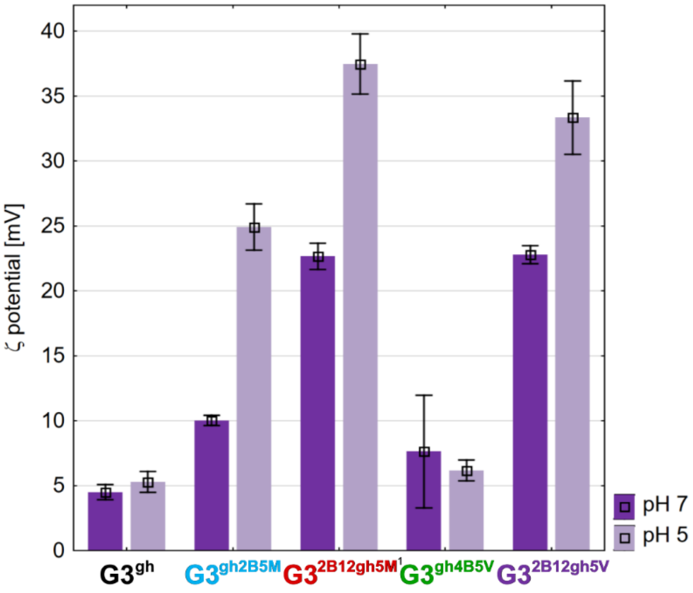

Figure S6. Zeta potential of conjugates measured in water (pH 7) and acetate buffer (pH 5). Data are presented as a mean ± standard deviation. [1] Values for G3^{2B12gh5M} were taken from [29].

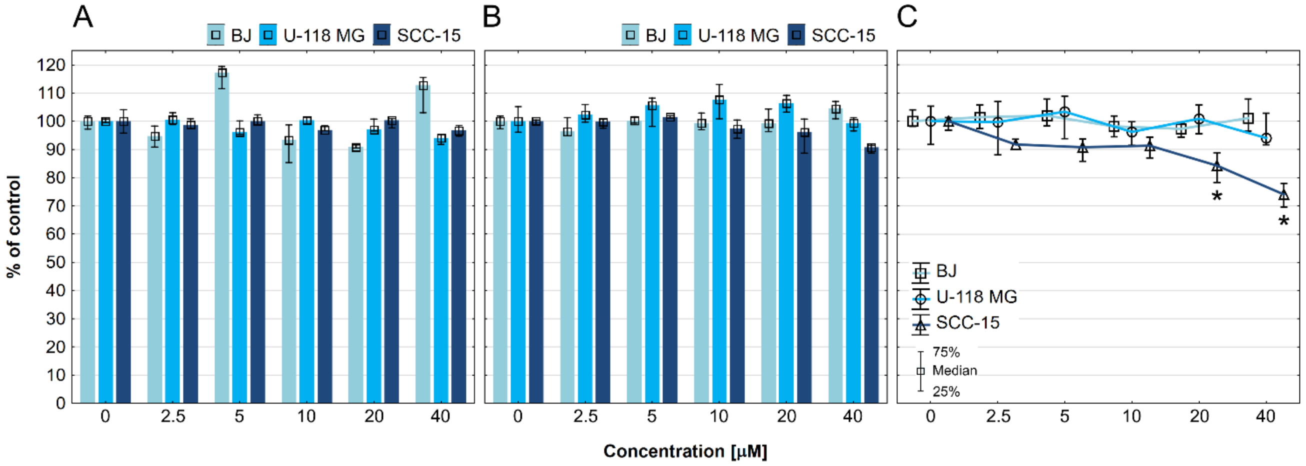

Figure S7. Cytotoxicity of **G3**^{gh} vehicle estimated with NR (**A**) and XTT (**B**) assay after 48 h incubation with normal fibroblasts (BJ), glioblastoma cells (U-118 MG) and squamous carcinoma cells (SCC-15). Anti-proliferative activity (**C**) of **G3**^{gh} against BJ, U-118 MG, and SCC-15 cells after 72 h of incubation, determined with Hoechst 33342 staining. Results are presented as medians of a percent against non-treated control (control expressed as 100%). The whiskers indicate lower (25%) and upper (75%) quartile ranges. * $p \leq 0.05$; Kruskal-Wallis test (against non-treated control).

Table S1. The ^{1}H and ^{13}C resonance assignments of the **G3**^{gh4B5V}, V, **G3**^{gh2B5M} and αM based on COSY and HSQC/HMBC spectra in DMSO-d_6. The superscripts: ^{B}, ^{gh}, ^{V} and ^{M} are related to Biotin, glucoheptoamide, Vadimezan and α-Mangostin, respectively. The annotation nd means not determined due to low intensity of resonances or overlapping with high intensity peaks from PAMAM.

Species / Locant	G3^{gh4B5V}		Vadimezan		G3^{gh2B5M}		α-Mangostin	
	^{1}H	^{13}C	^{1}H	^{13}C	^{1}H	^{13}C	^{1}H	^{13}C
$b_{0\text{-}3}$(PAMAM)	2.19	33.2			2.20	33.5		
$d_{0\text{-}2}$(PAMAM)	2.42	52.2			2.45	52.6		
$a_{0\text{-}3}$ (PAMAM)	2.65	49.5			2.66	49.9		
d_3′(PAMAM)	3.08	37.0			3.07	37.2		
$c_{0\text{-}3}$(PAMAM)	3.13	38.3			3.13	38.6		
-COO(G3)	-	171.5			-	171.9		
-COO(G3)	-	171.8			-	173.8		
NH(G3)	7.98				7.93			
2^B	2.05	35.3			nd	nd		
3^B, 4^B, 5^B	1.20-1.65	25.3			1.21	nd		
3^B, 4^B, 5^B	1.20-1.65	28.1						
3^B, 4^B, 5^B	1.20-1.65	28.3						
6^B	3.08	55.5			nd			
7^B	2.78	nd			nd			
8^B	4.31	59.4			4.30	nd		
9^B	4.13	61.2			4.12	nd		
10^B	6.40	nd			6.40	nd		
11^B	6.46	nd			6.48	nd		
12^B	-	163.0			-	nd		
1^{gh}	-	172.5			-	nd		
2^{gh}	3.94	72.2			3.94	72.4		
3^{gh}	3.69	74.6			3.69	74.8		
4^{gh}	3.85	68.1			3.86	68.3		
5^{gh} or 6^{gh}	3.46	73.4			3.45	73.7		
5^{gh} or 6^{gh}	3.48	71.6			3.48	71.8		
7^{gh}	3.60-3.30	63.3			3.60-3.30	63.5		
$4'^{gh}$, $5'^{gh}$, $6'^{gh}$, $7'^{gh}$	4.54-4.43	-			5.62-4.51	-		

3′[gh]	4.86	-				-		
2′[gh]	5.60	-				-		
1[V]	7.98	124.5	8.09	124.6				
2[V]	7.38	123.7	7.41	123.8				
3[V]	7.73	136.7	7.79	136.7				
4[V]	-	120.6	-	120.6				
5[V]	-	144.8	-	144.8				
6[V]	-	118.9	-	118.9				
7[V]	7.26	126.1	7.30	126.1				
8[V]	7.88	122.7	7.92	122.7				
9[V]	-	176.5	-	176.3				
10[V]	-	153.9	-	154.0				
11[V] or 12[V]	-	169.6	-	169.3				
13[V]	-	153.4	-	153.5				
14[V], 15[V]	2.37	20.3	2.41	11.15<br>20.25				
16[V]	nd	nd	3.98	35.7				
17[V]	-	173.6	-	172.0				
1[M]					-	159.9	-	160.0
1′[M]					13.73	-	13.72	-
2[M]					-	109.8	-	109.7
3[M]					-	162.6	-	162.9
3′[M]					-	-	11.01	-
4[M]					6.34	92.7	6.34	92.6
4a[M]					-	154.5	-	154.5
9a[M]					-	nd	-	154.9
9[M]					-	181.7	-	181.2
8a[M]					-	nd	-	108.0
10a[M]					-	155.1	-	155.8
5[M]					6.79	102.1	6.80	101.9
6[M]					-	157.3	-	161.6
6′[M]					-	-	10.82	-
7[M]					-	143.7	-	145.1
7′[M]					3.69	60.5	3.70	60.2
8[M]					-	136.7	-	135.6
11[M]					3.99	26.0	4.01	26.2
12[M]					5.15	124.1	5.16	124.8
13[M]					-	130.8	-	130.2
14[M]					1.76	18.6	1.77	18.4
15[M]					1.61	26.1	1.62	26.1
16[M]					3.19	21.4	3.21	21.6
17[M]					5.15	122.8	5.16	123.3
18[M]					-	130.9	-	130.6
19[M]					1.71	18.1	1.72	18.2
20[M]					1.61	26.0	1.62	26.0
